# 1235. A Retrospective Review of Antimicrobial Stewardship Audit and Feedback: Assessing Acceptance of Stewardship Recommendations During the COVID-19 Pandemic

**DOI:** 10.1093/ofid/ofad500.1075

**Published:** 2023-11-27

**Authors:** Melissa A Colaluca, Michelle Korson, Dora E Wiskirchen, Jessica Abrantes-Figueiredo

**Affiliations:** The University of Connecticut, Hartford, Connecticut; The University of Connecticut, Hartford, Connecticut; Saint Francis Hospital and Medical Center, Hartford, Connecticut; Saint Francis Hospital, Hartford, Connecticut

## Abstract

**Background:**

Antimicrobial stewardship is an underutilized strategy to mitigate the impact of antimicrobial resistance. It is well documented that many Antimicrobial Stewardship Programs (ASPs) were tasked with assisting healthcare teams in navigating the COVID-19 pandemic, leaving less time/fewer resources for day-to-day ASP activities. The aim of this study was to evaluate the acceptance of stewardship recommendations during a season when the ASP had less time to perform regular educational activities and handshake stewardship.

**Methods:**

A retrospective study of patients receiving antimicrobial therapy at Saint Francis Hospital during 12/30/2020-10/01/2022, for which audit and feedback by the ASP was conducted. A well-established ASP existed prior to the pandemic. Independent variables included the reason for intervention, type of infection by organ system, and type of intervention. Interventions included recommendations to stop antibiotics, de-escalation, duration of therapy, ID consultation, or change the route of administration. The primary outcome was the audit and feedback acceptance rate, and the secondary outcome was whether the intervention resulted in infectious disease consultation.

**Results:**

297 patients underwent audit and feedback. Interventions most accepted by providers included changing the route of administration (100%) and consulting ID formally (92%). Adjusting the duration of therapy was the least accepted intervention at 78%. Regardless of intervention, 33% of providers requested a formal ID consult. The number of interventions varied sporadically, coinciding with periods of decreased availability by the ASP team and during peak waves of the COVID-19 pandemic.

**Conclusion:**

Our ASP team, despite decreased resources while battling an ongoing pandemic, were still able to perform audit and feedback to providers with a good acceptance rate of recommendations, consistent with current literature. Having not only an existing but robust ASP prior to the pandemic, allowed continued success with ASP intervention acceptance rates, despite less dedicated time for typical ASP activities during the pandemic. Fostering good relationships between providers and ASP teams, as well as continued support and funding from hospital leadership, are vital to ongoing success.

**Disclosures:**

**All Authors**: No reported disclosures

